# Intra-articular morphine versus bupivacaine for knee motion among patients with osteoarthritis: randomized double-blind clinical trial

**DOI:** 10.1590/S1516-31802008000600003

**Published:** 2008-11-06

**Authors:** Miriam Bellini Gazi, Rioko Kimiko Sakata, Adriana Machado Issy

**Keywords:** Analgesia, Morphine, Bupivacaine, Osteoarthritis, Knee, Analgesia, Morfina, Bupivacaína, Osteoartrite, Joelho

## Abstract

**CONTEXT AND OBJECTIVE:**

Osteoarthritis causes pain and disability in a high percentage of elderly people. The aim of the present study was to assess the efficacy of intra-articular morphine and bupivacaine on the joint flexion and extension angles of patients with knee osteoarthritis.

**DESIGN AND SETTING:**

A randomized double-blind study was performed at a pain clinic of Universidade Federal de São Paulo.

**METHODS:**

Thirty-nine patients with pain for more than three months, of intensity greater than three on a numerical scale (zero to 10), were included. G1 patients received 1 mg (1 ml) of morphine diluted in 9 ml of saline, intra-articularly, and G2 patients received 25 mg (10 ml) of 0.25% bupivacaine without epinephrine. Pain was assessed on a numerical scale and knee flexion and extension angles were measured after administration of the drugs at rest and during movement. The total amount of analgesic supplementation using 500 mg doses of paracetamol was also determined.

**RESULTS:**

No significant difference in pain intensity was observed between G1 and G2. Significant decreases in pain at rest and during movement and significant increases in mean flexion and extension angles were observed in both groups, with no significant difference between the two groups. The mean total amount of paracetamol used over a seven-day period was 3578 mg in G1 and 5333 mg in G2 (P = 0.2355; Mann-Whitney test).

**CONCLUSION:**

The analgesic effects of 1 mg of morphine and 25 mg of 0.25% bupivacaine were similar among patients with osteoarthritis of the knee.

**CLINICAL TRIAL REGISTRATION NUMBER:**

NCT00636415.

## RESUMO

**CONTEXTO E OBJETIVO:**

A osteoartrite é a mais freqüente entre as doenças articulares em pessoas idosas. O objetivo do estudo foi avaliar a eficácia da bupivacaína e da morfina por via intra-articular no ângulo de flexão e extensão da articulação do joelho em pacientes portadores de osteoartrite.

**TIPO DE ESTUDO E LOCAL:**

Estudo aleatório e duplo–cego, realizado na Clínica de Dor.

**MÉTODOS:**

39 pacientes foram divididos em dois grupos: G1 (n = 18) recebendo 1 mg (1 ml) de morfina por via intra-articular diluída em 9 ml de soro fisiológico a 0,9% e G2 (n = 21) recebendo 25 mg (10 ml) de bupivacaína 0,25% sem vasoconstritor por via intra-articular. A intensidade da dor foi avaliada pela escala numérica ao repouso e ao movimento, após a injeção dos medicamentos. Foi avaliada a necessidade de complementação analgésica com paracetamol (500 mg).

**RESULTADOS:**

Dos 39 pacientes, 31 completaram o estudo. Não houve diferença significante da dor pela escala numérica ao repouso e ao movimento entre os dois grupos nos tempos estudados. Não houve diferença entre os dois grupos no tempo entre a administração da solução intra-articular e na necessidade de complementação analgésica. A dose média do paracetamol utilizada no primeiro dia da semana foi de 796 mg no G1 e de 950 mg no G2; a complementação na semana foi de 3578 mg no G1 e 5333 mg no G2.

**CONCLUSÃO:**

O efeito analgésico de 1 mg de morfina intra-articular e de 25 mg de bupivacaína a 0,25% sem vasoconstritor são semelhantes em pacientes com osteoartrite do joelho.

## INTRODUCTION

The main purpose of medical care is to preserve the patient's functional ability by properly diagnosing and managing diseases. Osteoarthritis, the most frequent joint disease, causes pain and disability in a large proportion of elderly individuals^1^ At present, and not only in developed countries, the elderly account for a large portion of the patients suffering from severe and difficult-to-control chronic pain. As a result, there is concern about worsened quality of life^1^ Osteoarthritis pain has a profound impact on elderly individuals, with several consequences such as depression, reduced activity, sleep pattern disruption and gait impairment.

Different organs are often affected in these patients, which restricts the number of drugs and analgesic techniques that can be used for pain relief. In addition, analgesics may interact with drugs that are used to control other diseases^1^

Osteoarthritis pain can be managed with different drugs and techniques. Even when administered in combination, adequate pain relief may not be achieved, and an intra-articular drug injection is then recommended^2^

Opioids and local anesthetic agents are often used for the treatment of acute pain. However, there are no controlled trials comparing the effect of these pharmaceutical agents on joint motion among patients with osteoarthritis.

## OBJECTIVE

The aim of the present study was to assess the efficacy of intra-articular morphine and bupivacaine on the joint flexion and extension angles of patients with knee osteoarthritis.

## METHOD

Thirty-nine patients over 50 years of age with a radiological confirmation of chronic knee osteoarthritis were enrolled in the study^3^ All of them had presented pain lasting for more than three months (either at rest or under strain), morning stiffness, absence of heat at the site, crepitation during movement and a pain score ranging from three to ten. The patients were selected with regard to their state of degeneration and restriction due to ankylosis or pain, as defined by a Kellgren and Lawrence score from two to four. We then made measurements of angles of movement.

Patients with coagulopathy, infection or malignant disease, patients who had undergone knee or hip surgery and patients who had been using opioids during the 24 hours prior to the study were excluded.

The study was conducted in a double-blind manner and each patient was randomly assigned to one of two groups. The process of randomization consisted of drawing lots for the procedures, which were held in sealed envelopes. The draw was performed by the nurse who prepared the medication. A physician was responsible for injection of the intra-articular medication and the researcher was responsible for evaluating the patients. Both the physicians and the patients were unaware of the group chosen by lot until the end of the study.

The medications were prepared in 10 ml syringes and the volume and color of the solutions were the same for the two groups. Thus, both the physician responsible for the procedure and the researcher were unaware of the group to which the patient belonged.

Group 1 patients (n = 18) received 1 mg (1 ml) of morphine diluted in 9 ml of saline, intra-articularly. Group 2 patients (n = 21) received 10 ml of 0.25% bupivacaine without epinephrine. For the intra-articular injection, the patient remained seated with flexed joints. After antisepsis, a puncture was performed between the medial patellar surface and the femoral condyle, and a 25 G needle was inserted through the skin and subcutaneous tissue, as far as the joint capsule within the joint. The analgesic was then injected slowly.

The following parameters were assessed: analgesia duration, pain intensity rated on a numerical scale (zero to 10), where zero corresponded to no pain and 10 to the worst pain, goniometer-based measurement of the joint angle during both flexion and extension (before infiltration and 30 minutes, 60 minutes and 7 days after drug administration), and the total dose of analgesic supplementation needed over seven days. In addition, the incidence of adverse effects was determined over the seven days of the study.

This was an efficacy study, so only the patients who concluded the study were evaluated. Among the 39 patients selected, two patients from group 1 (G1) and five from group 2 (G2) were excluded from some of the analyses because they did not return on the day of assessment or because they used a supplementary analgesic differing from the one standardized for this study.

The sample size calculation suggested that a group size of about 18 patients should provide a 95% chance (power of 95%) of detecting a difference at a significance level of 0.05. Parametric and nonparametric tests were used for statistical analysis on the results, depending on the nature of the variables studied. The following tests were applied: Mann-Whitney test for comparisons of age, gender, body mass index, radiological alterations and pain duration between groups; Student's t-test for weight and height; Kruskal-Wallis test for comparisons of knee flexion and extension angles at each time within the same group. Central trend (mean) and dispersion (standard deviation) measurements were used for statistical analysis. The significance level was set at P ≤ 0.05.

This study was approved by the institutional Ethics Committee and written consent was obtained from all patients.

## RESULTS

Out of the 39 patients (G1, n = 18; G2, n = 21) that took part in this study, two were excluded from G1 and another two from G2 on the seventh day because either they did not return on the evaluation date or they used supplementary analgesic that differed from what was prescribed for this study. Out of the 21 patients in G2, seven were excluded from the evaluations of pain intensity, flexion and extension angle, analgesia quality and side effects because five had used rescue medication that differed from what was indicated in the protocol and two did not return on the seventh day. Nevertheless, for one of these seven excluded patients, the data from evaluations of time elapsed between solution administration and supplementary analgesic and the data on the paracetamol doses used on the first and seventh days were included in the study because this patient's pain diary was correctly filled out.

The demographic data (gender, age, weight, height and body mass index) of the 39 patients were similar in the two groups (Table 1).

**Table 1:** Patients’ demographic data

	Gender	Age	Weight	Height	BMI
	(M, F)	(years)	(kg)	(cm)	(kg/m^2^)
G1 (n = 18)	17; 1	67.6 ± 10.5 (62.3 – 72.8)	80.8 ± 15.5 (73.1 – 88.5)	156.4 ± 5.6 (153.6 – 159.2)	32.0 ± 5.0 (29.6 – 36.6)
G2 (n = 21)	20; 1 (0.06 – 20.2)	64.3 ± 11.3 (59.2 – 69.4)	74.3 ± 9.3 (70.0 – 78.8)	157.9 ± 7.6 (154.5 – 161.4)	29.7 ± 3.6 (28.0 – 31.3)
P	1.000*	0.3824†	0.2366‡	0.5824‡	0. 2046†

*Data are reported as mean ± standard deviation (95% confidence interval). G1 = intra-articular morphine; G2 = intra-articular bupivacaine; P ≤ 0.05 = statistically significant; M = male; F = female; BMI = body mass index;*

* *Fisher's exact test;*

† *Mann-Whitney test;*

‡ *Student's t-test.*

No differences in radiological abnormalities were observed between the two groups. Pain duration ranged from four months to 40 years and was similar in the two groups (group 1: 89.2 months, and group 2: 113.8 months; Mann-Whitney test).

All patients in the two groups had previously taken analgesic drugs. In group 1 (n = 18), 44.5% of the patients had used these drugs for three months to one year, 50.0% for one to 10 years, and 5.5% for 10 to 20 years. In group 2 (n = 21), the duration of analgesic use ranged from three months to one year for 23.9% of the patients and from one to 10 years for 76.1%.

Flexion and extension angles were assessed on day 7 for 16 patients in group 1 (two did not return), and for 14 in group 2 (four used analgesics other than those standardized in the protocol and three did not return). No significant differences in the mean flexion angles at the established time points (T_0_, T_30_, T_60_, T_7_) were observed between the two groups (Mann-Whitney test). The mean flexion angles increased over time (T_0_, T_30_, T_60_ and T_7_) in both groups (Kruskal-Wallis test) (Table 2). No significant differences in the mean extension angles were observed between the two groups at the time points analyzed (T_0_, T_30_, T_60_ and T_7_; Mann-Whitney test). The mean extension angles increased over time (T_0_, T_30_, T_60_ and T_7_) in both groups (Kruskal-Wallis test) (Table 3).

**Table 2:** Knee flexion angle

	Time				
	T_0_	T_30_	T_60_	T_7_	ANOVA
G1 (n = 18)	108.9 ± 9.2 (104.3 – 113.4)	114.4 ± 8.9 (110.0 – 118.8)	115.9 ± 8.5 (111.7 – 120.2)	115.9 ± 8.4* (111.4 – 120.4)	0.0491
G2 (n = 21)	107.6 ± 7.2 (104.3 – 110.9)	114.7 ± 8.4 (110.9 – 118.6)	116.4 ± 10.0 (111.0 – 120.3)	113.6 ± 9.7† (107.9 – 119.1)	0.0168
Mann-Whitney	0.8090	0.8986	0.8590	0.4783	

*Data are reported as mean ± standard deviation (95% confidence interval). G1 = intra-articular morphine; G2 = intra-articular bupivacaine; T_0 =_ immediately before drug administration; T_30_ = 30 minutes; T_60_ = 60 minutes; T_7_ = 7 days after drug administration;*

* *G1 (n = 16);*

† *G2 (n = 14); P ≤ 0.05= statistically significant; ANOVA = analysis of variance.*

**Table 3:** Knee extension angle

	Time				
	T_0_	T_30_	T_60_	T_7_	ANOVA
G1 (n = 18)	5.27 ± 6.05 (2.2 – 8.3)	2.22 ± 3.91 (0.2 – 4.1)	1.38 ± 3.34 (-0.2 – 3.0)	1.25 ± 3.41* (-0.5 – 3.0)	0.0448
G2 (n = 21)	3.81 ± 4.4 (1.7 – 5.8)	1.19 ± 3.12 (-0.2 – 2.6)	0.95 ± 2.55 (-0.2 – 2.1)	1.42 ± 3.05† (-0.3 – 3.1)	0.0330
Mann-Whitney	0.5677	0.4719	0.8709	0.7459	

*Data are reported as mean ± standard deviation (95% confidence interval). G1 = intra-articular morphine; G2 = intra-articular bupivacaine; T_0_ = immediately before drug administration; T_30_ = 30 minutes; T_60_ = 60 minutes; T_7_ = 7 days after drug administration;*

* *G1 (n = 16);*

† *G2 (n = 14); P ≤ 0.05 = statistically significant; ANOVA = analysis of variance.*

No significant difference in pain intensity evaluated using the numerical pain score at rest or under strain was observed between the two groups at the time points analyzed (T_0_, T_30_, T_60_ and T_7_; Mann-Whitney test) (Table 4).

**Table 4:** Pain intensity at rest and during movement

Time	At rest			During movement		
	G1 (n = 18)	G2 (n = 21)	P	G1 (n = 18)	G2 (n = 21)	P
**T**_0_	1.38 ± 1.97	1.72 ± 2.48	0.80	7.44 ± 2.03	7.00 ± 2.19	0.50
**T**_30_	0.44 ± 1.09	0.28 ± 0.95	0.71	3.11 ± 3.16	2.42 ± 2.29	0.63
**T**_60_	0.33 ± 0.97	0.14 ± 0.65	0.73	2.83 ± 2.40	2.04 ± 1.91	0.35
**T**_7_	0.37 ± 1.87*	0.92 ± 1.38†	0.35	3.81 ± 2.48*	4.00 ± 2.57†	0.88

*Data are reported as mean ± standard deviation (95% confidence interval). G1 = intra-articular morphine; G2 = intra-articular bupivacaine; T_0_ = immediately before drug administration; T_30_ = 30 minutes; T_60_ = 60 minutes; T_7_ = 7 days after drug administration;*

* *G1 (n = 16);*

† *G2 (n = 14); P ≤ 0.05 = statistically significant (Mann-Whitney test).*

There was no difference in the interval between intra-articular administration of the analgesic solution and the need for first supplementation of analgesic, which was 593.7 ± 389.8 minutes following morphine administration (group 1) and 733.3 ± 371.6 minuntes following bupivacaine (group 2) (P = 0.239, Mann-Whitney test) (Table 5).

**Table 5:** Time elapsed until first supplementation of analgesic

Groups	Time (minutes)
G1 (n = 16)	593.7 ± 389.8 (386.0 – 801.4)
G2 (n = 15)	733.3 ± 371.6 (527.5 – 939.1)
P	0.2399

*Data are reported as mean ± standard deviation (95% confidence interval). G1 = intra-articular morphine; G2 = intra-articular bupivacaine; n = number of patients; P ≤ 0.05 = statistically significant (Mann-Whitney test).*

The paracetamol dose needed for supplementation was 795.6 ± 1444.1 mg on the first day after intra-articular morphine administration (group 1; n = 16) and 949.7 ± 1318.0 mg after bupivacaine (group 2, n = 15), with no difference between the groups (Mann-Whitney test, P = 0.452). The total dose of paracetamol used over a one-week period was 3578.1 ± 2885.1 mg in group 1 (n = 16) and 5333.3 ± 3782.8 mg in group 2 (n = 15), with no difference between the groups (P = 0.2355, Mann-Whitney test).

Near-syncope was observed in 12.5% of group 1 patients, five hours after infiltration, and 6.66% of group 2 patients presented vomiting three hours after infiltration. No complications were observed during infiltration in either group.

## DISCUSSION

Osteoarthritis is clinically characterized by inflammation, pain and joint stiffness. Although osteoarthritis is mainly a joint disease, it is usually managed with systemic nonsteroidal anti-inflammatory drugs. However, these drugs are of limited use because of gastrointestinal or renal side effects, especially in older populations in which the incidence of osteoarthritis is higher. In addition, interactions may occur with other drugs concomitantly taken for the chronic diseases prevalent in this age group.

Administration of opioid or local anesthetics directly into the knee joint can promote adequate pain control without causing the side effects observed for anti-inflammatory agents administered through a systemic route. The analgesic response observed in the present study after intra-articular administration of an opioid or local anesthetic may contribute towards encouraging regular physical exercise therapy. Such therapy has the aims of reducing limitations and improving joint function by increasing motion range and muscle strength, thus improving both balance and the execution of daily activities^4^

Several studies on acute pain have assessed intra-articular morphine and bupivacaine^5-7^ Other investigations have been conducted to determine the peripheral mechanism of action of opioids^8^ However, few studies analyzing intra-articular injection in patients with chronic knee pain are available. One study showed increased motion range after administration of 1 mg of morphine combined with 10 ml of 0.25% bupivacaine in five patients^9^ Stein et al^10^ compared intra-articular morphine and dexamethasone over a 24-hour period. Creamer et al^11^ compared the analgesic effect of intra-articular injection of 5 ml of 0.25% bupivacaine and placebo after 24 hours and 7 days. However, no studies comparing intra-articular morphine and bupivacaine in patients with chronic pain are available.

The present sample mainly consisted of women, which is in line with the literature, in which higher prevalence of osteoarthritis among women over the age of 45 years is shown^12^ Obesity has been reported to be one of the major risk factors for osteoarthritis, through aggravating the pain condition^13^ In the present study, the patients in the two groups presented high body mass index, thus characterizing an obese population. However, no difference in the demographic data that would explain the variations in the pharmacological action of the medications used was observed between the two groups.

A correlation was observed between the imaging severity score and pain intensity, although the literature shows that, even in the absence of radiological alterations in the knee, approximately 31% of osteoarthritis patients complain of pain^2,12^ Most patients had a radiological severity score of 3 or 4, thus indicating the importance of this score for determining disease severity. When combined with physical examination and evaluation of joint function, this score can be used to establish the progression of the disease.

Inadequately controlled, persistent and long-lasting pain results in impairment of neuroplasticity. This condition causes patients to be in pain not only during movement but also at rest, with consequent limitations on joint functional ability and activities of daily life^14,15^ In the present study, the patients presented long-term pain, in keeping with other reports^1^ Our study confirms the fact that analgesics are used over long periods to control persistent pain: nonsteroidal anti-inflammatory drugs, either medically or self-prescribed, were the most widely used agents. Opioid analgesics were used less frequently and for short periods of time, probably due to the reluctance of professionals to prescribe opioid medication, which is in agreement with another investigation^1^

Most of the present patients had concomitant diseases. Other studies have suggested that the high prevalence of knee osteoarthritis in patients with diabetes mellitus and high blood pressure and a high mortality rate is due to chronic-degenerative conditions^1^

Measurement of the angle of motion during knee flexion or extension before and after infiltration is the parameter used to assess the limit imposed not only by anatomical abnormalities in joints but also by pain^16^ In the present study, the joint angle was measured objectively with a goniometer before and after administration of each medication at different times. One study reported a greater angle of motion after intra-articular infiltration of 25 mg of bupivacaine and 1 mg of morphine for chronic knee pain, although no objective measurements were made^17^ In our study, increases in these angles were observed in both groups after injection, thus showing that both morphine and bupivacaine provide pain relief effects.

The patients in both groups received the analgesic solutions intra-articularly. These analgesic solutions were identical in volume and appearance, in the same way as already reported in another study,^7^ in order to reduce the chance of bias when assessing the level of pain relief.

The use of a 1-mg dose of intra-articular morphine was in accordance with procedures used in another study^6^ that reported that this gave rise to 24-hour analgesia after arthroscopy of the knee. The relationship between the intra-articular opioid dose and outcome has not yet been established. Several doses have been used, with no convincing additional effects on analgesia quality or duration that would recommend the use of higher doses^6,18^ The volume of solution injected into the intra-articular space is still a controversial issue and varies among published studies^9,17,19^

The analgesic effect following injection was also assessed based on the need for first supplementation of analgesic, as well as on the total amount of analgesic consumed during the study period, as also reported in other studies^6^

A decline in the number of patients was observed in both groups after one week because some patients did not return or they failed to bring their daily pain record. Some of them possibly did not return because they had achieved adequate pain relief.

Our study showed that morphine (1 mg) and bupivacaine promoted pain relief at rest and under strain. After one week, the pain intensity was similar in the two groups. However, analgesic supplementation was required and the patients used different amounts of paracetamol.

Over the same period, some patients in the two groups still presented good analgesia. However, this effect was probably due not only to the intra-articular injection, but also to analgesic supplementation with paracetamol.

After injection, the joint angles were found to be greater in both groups, thus demonstrating that both morphine and bupivacaine provide analgesic effects that lead to improved joint function.

## CONCLUSION

A similar analgesic effect was observed for 1 mg of intra-articular morphine and 25 mg of 0.25% bupivacaine in patients with knee osteoarthritis.
